# Yao herbal medicinal market during the Dragon Boat Festival in Jianghua County, China

**DOI:** 10.1186/s13002-018-0260-5

**Published:** 2018-10-17

**Authors:** Binsheng Luo, Yujing Liu, Bo Liu, Sizhao Liu, Beixi Zhang, Linghan Zhang, Chunrui Lin, Yan Liu, Edward J. Kennelly, Zhiyong Guo, Chunlin Long

**Affiliations:** 1 Key Laboratory of Ethnomedicine (Minzu University of China), Ministry of Education, Beijing, 100081 China; 2 College of Life and Environmental Sciences, Minzu University of China, Beijing, 100081 China; 3 Key Laboratory of Modern Agricultural Equipment and Technology, Ministry of Education, Jiangsu University, Zhenjiang, 212013 Jiangsu China; 4 Guangxi Institute of Botany, Guangxi Region and Chinese Academy of Sciences, Guilin, 541006 China; 5 Lehman College, City University of New York, Bronx, New York, 10468 USA; 6 Kunming Institute of Botany, Chinese Academy of Sciences, Kunming, 650201 China

**Keywords:** Dragon Boat Festival, Yao ethnic group, Jianghua County, Traditional knowledge, Conservation

## Abstract

**Background:**

The traditional medicinal markets held during the Dragon Boat Festival are common and important in China’s countryside. In Jianghua, a Yao autonomous county in Hunan Province in China, the medicinal market also plays an important role for the application, conservation, and communication of traditional Yao medicinal knowledge.

**Methods:**

During the Dragon Boat Festival in 2016 and 2017, ethnobotanical surveys and inventories were conducted in the medicinal market of Jianghua County, and voucher plant specimens were collected, identified, and deposited in a herbarium. Quantitative analysis included measurement of frequency of occurrence for species in the marketplace and the relative importance index for the number of uses for a given species.

**Results:**

A total of 306 plant species (249 genera, 113 families) and their related information about the medicinal market were collected. Some major findings include the following: (1) Using the whole plant as medicine is more common than other medicinal plant parts; (2) treating rheumatism and clearing inner heat are the most frequent medicinal uses; and (3) taking a medicinal bath is the most frequent modality to administer the traditional medicine. The frequency of occurrence and the relative importance index of some medicinal plants were analyzed, as well as the demographics and the number of stalls and the status of traditional Yao medicinal knowledge in Jianghua. Based on the investigation, suggestions were proposed for better protecting the medicinal market and preserving traditional medicinal knowledge in Jianghua County.

**Conclusion:**

The medicinal market during the Dragon Boat Festival in Jianghua County possesses an important cultural value and helps to conserve the traditional Yao medicinal knowledge. The medicinal plants sold at the market showed great diversity and unique local characteristics. The medicinal market is facing some challenges in such a rapidly developing era. Cultivation of young healers and maintaining the local biodiversity might be the key solutions for the development of local medicinal market and local Yao medicinal knowledge.

## Background

The Dragon Boat Festival, occurring on the fifth day of the fifth month in the Chinese lunar calendar, is one of the most famous traditional festivals in China. People eat *zongzi* (a special food made from sticky rice and other ingredients), drink realgar wine, and race dragon boats to celebrate this festival all over the country. However, in Jianghua, a county with the largest population of Yao people in China [1], the Dragon Boat Festival is a special opportunity for the local people to trade medicinal plants in a large market. It has become the most important tradition in Jianghua. During this festival, the Yao villagers bring medicinal plants collected recently to the market. They share and exchange the experiences of identifying, harvesting, and applying their medicinal plants with each other and with consumers. This unique medicinal market has already become a great platform for different people to communicate with and learn from each other [2]. This spontaneous traditional activity is also making vital contributions to the sustainable conservation, transmission, and expansion of related traditional knowledge [3].

The Yao is an ancient ethnic group, and one of the 55 officially recognized minority groups of the Chinese government. The largest populations of Yao live in the mountains and high ranges of southern China and practice slash-and-burn agriculture and hunt [4, 5]. Based on the long-term practice, the Yao people depend on local plant resources to prevent and treat diseases. They have developed their own traditional medicine system, as well as distinct customs to promote health. For example, they use *Acorus calamus*, *Artemisia argyi*, and realgar to keep pests and pathogens away [4]. Our previous investigation (unpublished) indicated that in traditional culture of the Yao ethnic group, the Dragon Boat Festival is believed to be the birthday of the so-called Medicinal Lord. The effect of medicinal plants during this festival is believed to be the best by local people. Thus, the medicinal market has become the biggest and the most popular event on the Dragon Boat Festival in Jianghua.

In recent years, more scientists have studied natural herbal medicine to determine their efficacy and potentially develop validated new drugs and health care products [5, 6]. As a natural treasury of traditional medicinal knowledge, the markets selling herbal drugs possess great potential for new drug discovery [7]. Using the Web of Science with search term “medicinal market and China”, only four English-language research papers can be found [8–11].

As a cultural phenomenon in China, several Chinese-language papers have reported different medicinal markets during the Dragon Boat Festival, such as the investigations in Jingxi County [2, 12], Yongzhou City [13], and Gongcheng County [14]. These studies showed the species diversity of medicinal plants and their medicinal parts, medicinal purposes, modalities, and other information [2, 12–14]. Much of the traditional knowledge is in danger of being lost, so these local medicinal plant resources need to be protected [2, 12–14]. In Jianghua, the medicinal market in the Dragon Boat Festival is relatively large in scale, but very little scientific research has been carried out [15, 16].

Nowadays, as much traditional knowledge is in danger of disappearing, the traditional knowledge associated with the Jianghua medicinal marketplace should be preserved. Therefore, an ethnobotanical research focused on Jianghua medicinal market was conducted at the Dragon Boat Festival in 2016 and 2017. This study evaluates the status of the Jianghua medicinal market and analyzes the relationship among this medicinal market, local community, and local natural environment. Based on the study results, some suggestions are included for local communities to protect this medicinal market. Furthermore, this study may provide valuable clues for future development and also give comprehensive and scientific guidance for local people to consume the medicinal herbs in a safer manner.

## Methods

### Study site

**Fig. 1:**
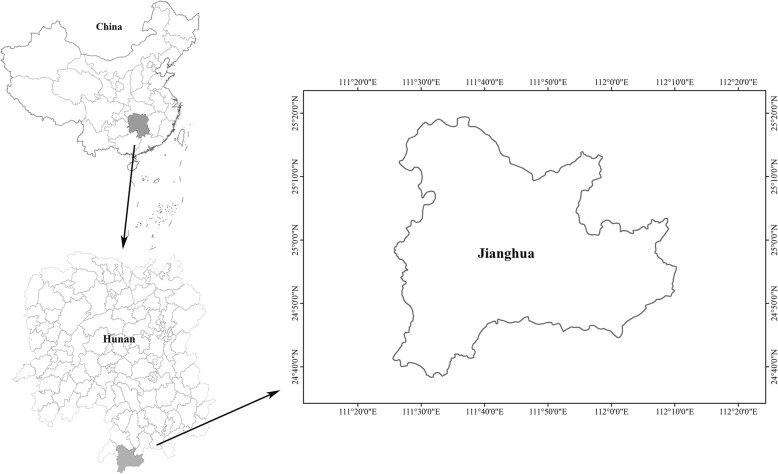
The location of Jianghua County, Hunan Province, China

Jianghua Yao Autonomous County belongs to Hunan Province and is located close to the border area of Guangdong Province, Guangxi Region, and Hunan Province in South China (Fig. 1). This area has a rich biodiversity, plentiful rainfall, and a mild temperature due to the low-latitude subtropical monsoon climate [17]. The population of Jianghua County is predominated by Yao people who account for more than half of the county’s population. Other ethnic groups like Zhuang, Han, and Miao also live there but have smaller populations [17]. Our previous investigation showed that the medicinal market distributes on Changzhen Street and its branches. The market starts 2 days before the Dragon Boat Festival and it grows to its largest on the festival day.

### Ethnobotanical data collection and statistical analysis

An ethnobotanical method was mainly used for this study. At the local market, each stall and vendor was investigated, and relevant information was recorded for all of the medicinal plants in trade. The chosen informants were vendors, buyers, and folk healers as well as other old knowledgeable people. Key informant interview was comprised of semi-structured interview and free listing based on the informant consensus. By means of different interview methods, comprehensive information about the medicinal plants in the market for further analysis was obtained.

Quantitative analysis was used to reveal the taxonomic characters and diversity of the modalities, using parts and medicinal uses of the medicinal herbs. The medicinal market opened around the Dragon Boat Festival which is only about 3 days each year. We intensively collected information from the medicinal market (269 stalls), and the methods including pairwise comparison and rank ordering are almost impossible even they are much more robust. Instead, the frequency of the occurrence and the relative importance (RI) index of the medicinal herbs were employed.

RI was originally proposed by Bennet et al. in 2000 [18]. This index is used to evaluate the degree of development and utilization of certain plant species. The formula of RI is as below [19]:

$$ \mathrm{RI}=\mathrm{NUT}+\mathrm{NT} $$

NUT is the number of categories used for a certain species divided by the number of all categories, and NT is the number of types of uses of a specific species divided by the number of all use types. During this study, NUT was equated as the number of types of therapeutic modalities (NM) of a given species divided by the number of all modalities [19]. Thus, RI is the sum of the NM and NT as the following formula:

$$ \mathrm{RI}=\mathrm{NM}+\mathrm{NT} $$

The number of the vendors, the gender, and the age composition of the vendors were also analyzed.

Voucher specimens of medicinal plants were collected with assistance from the local people in the market, villages nearby, and local ecosystems. They were identified by botanical experts, Profs. Chunlin Long, Chunrui Lin, and Yan Liu and Dr. Bo Liu. All specimens of medicinal plants collected in Jianghua were deposited in the College of Life and Environmental Sciences, Minzu University of China. The information compiled includes the following: vernacular names, scientific names, taxonomic status, using parts, medicinal uses, modalities, and voucher numbers of all medicinal plants collected in Jianghua. All the medicinal plants and related information are shown in Table 1.

**Table 1:** Inventory of medicinal plants traded in the Jianghua medicinal market

Scientific name	Family name	Local name	Purposes	Usage	Part used	Voucher number	RI value
Gymnosperma							
*Cunninghamia lanceolata* (Lamb.) Hook.	Taxodiaceae	Cong Liang	Skin disease	Medicinal bath		JH-114	2
*Juniperus chinensis* L.	Cupressaceae		Treating rheumatism, promoting blood circulation, skin disease	Medicinal bath	Branch, leaf	JH-043	4
*Cephalotaxus fortunei* Hook.	Cephalotaxaceae		Treating cancer, treating rheumatism	Medicinal bath	Branch, leaf	JH-159	3
*Gnetum parvi+281:286folium (Warb.) W.C.Cheng*	Gnetaceae		Skin disease	Herbal tea, medicinal bath	Whole plant	JH-181	3
Angiospermae							
*Illicium verum* Hook.f.	Schisandraceae		Nourishing, relieving pain	Spices	Fruit	JH-027	3
*Kadsura coccinea* (Lem.) A. C. Sm.	Schisandraceae	Da Zuan	Relieving pain, treating rheumatism, promoting blood circulation	Medicinal bath, making tincture	Root, stem	JH-070	5
*Kadsura longipedunculata* Finet & Gagnep.	Schisandraceae	Xiao Zuan Gu Feng	Treating rheumatism, promoting blood circulation	Medicinal bath	Root, stem	JH-165	3
*Houttuynia cordata* Thunb.	Saururaceae	Ge Le Tao	Heat clearing and detoxifying, treating respiratory disease, treating heatstroke	Food, herbal tea, medicinal bath	Root, leaf	JH-089	6
*Saururus chinensis* (Lour.) Baill.	Saururaceae	Yi Bai Liang Bai	Treating gynopathy, skin disease	Herbal tea, food, medicinal bath, stewing	Whole plant	JH-061	6
*Piper betle* L.	Piperaceae		Heat clearing and detoxifying, diminishing inflammation, skin disease, treating cold	Decoction, medicinal bath	Stem, leaf	JH-029	6
*Asarum sagittarioides* C. F. Liang	Aristolochiaceae	Shan Ci Gu	Treating snake bite, treating rheumatism, relieving pain, traumatic injury	Medicinal bath, decoction	Whole plant	JH-277	6
*Fissistigma oldhamii* (Hemsl.) Merr.	Annonaceae	Xiang Teng	Treating rheumatism, relieving pain, strengthening muscles and bones	Medicinal bath	Root, stem	JH-180	4
*Fissistigma polyanthum* (Hook. f. & Thoms.) Merr.	Annonaceae	Xie Di Feng	Treating rheumatism	Herbal tea, medicinal bath	Whole plant	JH-298	3
*Cinnamomum camphora*(L.) Presl	Lauraceae	Zhang Shu Ye	Treating rheumatism, expelling parasite	Medicinal bath		JH-208	3
*Cinnamomum glanduliferum* (Wall.) Meisn.	Lauraceae		Treating rheumatism	Herbal tea, medicinal bath	Bark, root	JH-088	3
*Cinnamomum wilsonii* Gamble	Lauraceae	Gui Shu Pi	Treating rheumatism, treating arthritis	Medicinal bath, food	Bark	JH-077	4
*Lindera glauca* (Sieb. et Zucc.) Blume	Lauraceae	Jia Si Feng	Treating rheumatism, detoxifying, relaxing tendons and activating collaterals	Medicinal bath	Branch, leaf	JH-233	4
*Litsea cubeba* (Lour.) Pers.	Lauraceae		Treating rheumatism, promoting blood circulation, relieving pain, treating gastrointestinal disease	Food (fruit, bud)	Whole plant, fruit, bud	JH-153	5
*Chloranthus fortunei* (A. Gray) Solms	Chloranthaceae	Si Ji Feng	Treating rheumatism, treating cold, detoxifying, relieving cough	Medicinal bath, decoction	Whole plant	JH-055	6
*Sarcandra glabra* (Thunb.) Nakai	Chloranthaceae	Jiu Jie Cha	Treating rheumatism, promoting blood circulation, heat clearing and detoxifying	Leaf:medicinal bath; root: making tincture	Whole plant	JH-096	5
*Acorus calamus* var. *angustatus* Besser	Acoraceae	Yan Chang Pu	Skin disease, treating cold	Herbal tea, medicinal bath	Whole plant	JH-202	4
*Acorus calamus* L.	Acoraceae	Sha Jiang	Nourishing	Medicinal bath, food	Rhizome	JH-221	3
*Arisaema decipiens* Schott	Araceae		Treating rheumatism, promoting blood circulation	Medicinal bath	Root, stem	JH-295	3
*Pothos chinensis* (Raf.) Merr.	Araceae		Treating rheumatism	Herbal tea, medicinal bath	Whole plant	JH-185	3
*Potamogeton lucens* L.	Potamogetonaceae		Treating infantile malnutrition	Food	Whole plant	JH-184	2
*Dioscorea opposita* Thunb.	Dioscoreaceae		Nourishing, eliminating phlegm	Food	Tuber	JH-275	3
*Tacca plantaginea* (Hance) Drenth	Dioscoreaceae	Xia Zi Cao	Heat clearing and detoxifying, eliminating inflammation, stopping bleeding	Herbal tea, food, medicinal bath	Rhizome	JH-011	5
*Stemona tuberosa* Lour.	Stemonaceae		Treating respiratory disease, expelling parasite	Decoction	Tuber	JH-281	3
*Paris polyphylla* Sm.	Melanthiaceae	Du Jiao Lian	Heat clearing and detoxifying, relieving cough	External use, decoction	Rhizome	JH-260	4
*Disporum cantoniense* (Lour.) Merr.	Colchicaceae	Yao Bian Zhu	Relieving cough, promoting digestion	Decoction	Rhizome	JH-214	2
*Smilax china* L.	Smilacaceae	Niu Wei Cai	Treating rheumatism, detoxifying, promoting blood circulation	Root: food (stewing with chicken); leaf: medicinal bath	Rhizome	JH-246	5
*Smilax riparia* A. DC.	Smilacaceae	Da Sheng Jin	Treating rheumatism, relieving cough	Medicinal bath	Root, rhizome	JH-097	3
*Aletris spicata* (Thunb.) Franch.	Liliaceae	Jin Xian Diao Bai Mi	Nourishing, relieving cough, expelling parasite	Decoction, food	Whole plant	JH-178	5
*Anemarrhena asphodeloides* Bunge	Liliaceae	Fen Tiao Cai	Treating gastrointestinal disease, treating gynopathy	Decoction	Rhizome	JH-113	3
*Aspidistra elatior* Blume	Liliaceae	Wu Gong Gen	Nourishing, promoting blood circulation, relieving cough	Decoction	Rhizome	JH-174	4
*Aspidistra retusa* K.Y. Lang et S. Z. Huang	Liliaceae	Guo Shan Wu Gong	Nourishing, promoting blood circulation, relieving cough	Decoction	Rhizome	JH-130	4
*Liriope muscari* (Decne.) L. H. Bailey	Liliaceae		Nourishing	Decoction	Tuber	JH-271	2
*Ophiopogon bodinieri* H. Lév.	Liliaceae	Jiu Cai Mai Dong	Heat clearing	Decoction, medicinal bath	Tuber	JH-069	3
*Ophiopogon japonicus* (Thunb.) Ker Gawl.	Liliaceae	Qing Pi Cao	Nourishing	Herbal tea	Tuber	JH-217	2
*Reineckia carnea* (Andrews) Kunth	Liliaceae		Heat clearing, relieving cough	Decoction	Whole plant	JH-251	3
*Bulbophyllum odoratissimum* (J.E.Smith) Lindl.	Orchidaceae	Shi Xian Tao	Treating respiratory disease, treating infantile malnutrition, relaxing tendons and activating collaterals, eliminating inflammation	Herbal tea	Whole plant	JH-264	5
*Bulbophyllum pectinatum* Finet	Orchidaceae	Shi Shan Tao	Traumatic injury, treating respiratory disease, relieving cough	Decoction	Whole plant	JH-041	4
*Dendrobium catenatum* Lindl.	Orchidaceae		Relieving stomachache	Herbal tea	Stem	JH-265	2
*Dendrobium nobile* Lindl.	Orchidaceae		Treating diabetes, improving eyesight, nourishing, promoting gastrointestinal functions	Herbal tea	Stem	JH-101	5
*Galeola lindleyana* (Hook.f. & Thomson) Rchb.f.	Orchidaceae	Zou Ma Feng	Treating rheumatism, relieving headache	Decoction, making tincture	Whole plant	JH-058	4
*Luisia morsei* Rolfe	Orchidaceae	Diao Lan	Treating rheumatism, treating respiratory disease, treating cold, treating cancer	Decoction	Whole plant	JH-133	5
*Pholidota chinensis* Lindl.	Orchidaceae		Heat clearing and detoxifying, treating infantile malnutrition	Food (stewing with meat)	Pseudobulb	JH-146	3
*Spiranthes sinensis* (Pers.) Ames	Orchidaceae		Nourishing, detoxifying	Herbal tea	Whole plant	JH-122	3
*Gladiolus × gandavensis*	Iridaceae		Diminishing inflammation, traumatic injury, heat clearing and detoxifying	External use	Rhizome	JH-040	4
*Iris confusa* Sealy	Iridaceae		Diminishing inflammation, treating infantile malnutrition, treating respiratory disease	Medicinal bath	Rhizome	JH-193	4
*Dianella ensifolia* (L.) DC.	Asphodelaceae		Detoxifying, promoting blood circulation, relieving pain	External use	Whole plant	JH-282	4
*Hemerocallis citrina* Baroni	Asphodelaceae		Heat clearing and detoxifying, nourishing	Food (stewing with meat, flower), decoction (root)	Root, flower	JH-090	4
*Curculigo orchioides* Gaertn.	Amaryllidaceae		Treating rheumatism, nourishing, strengthening muscles and bones	Medicinal bath	Rhizome	JH-213	4
*Polygonatum sibiricum* F. Delaroche	Asparagaceae		Nourishing	Decoction, medicinal bath	Rhizome	JH-236	3
*Murdannia keisak* (Hassk.) Hand.-Mazz.	Commelinaceae		Heat clearing and detoxifying, inducing diuresis, treating snake bite	Decoction	Whole plant	JH-093	4
*Bulbophyllum odoratissimum* (Sm.) Lindl. ex Wall.	Musaceae		Treating heart disease	Herbal tea	Flower	JH-006	2
*Alpinia chinensis* (Retz.) Roscoe	Zingiberaceae	Jian Gan Feng	Treating rheumatism	Medicinal bath	Whole plant	JH-196	2
*Alpinia galanga* (L.) Willd.	Zingiberaceae		Treating rheumatism, nourishing	Medicinal bath	Fruit, rhizome	JH-046	3
*Alpinia japonica* (Thunb.) Miq.	Zingiberaceae	Huang Qi	Treating rheumatism, nourishing, relieving pain	Medicinal bath	Root, stem	JH-138	4
*Amomum villosum* Lour.	Zingiberaceae	Jing Gan Feng	Treating rheumatism, nourishing	Medicinal bath, making tincture	Fruit	JH-195	4
*Curcuma longa* L.	Zingiberaceae		Relieving pain, treating gynopathy, inducing diaphoresis	Spices: stewing with chicken	Rhizome	JH-128	4
*Typha orientalis* C. Presl	Typhaceae	Shui La Zhu	Nourishing	Medicinal bath	Flower	JH-134	2
*Juncus effusus* L.	Juncaceae	Shui Deng Xin	Heat clearing, inducing diuresis, treating respiratory disease, relieving cough,	Herbal tea	Stem pith	JH-262	5
*Imperata cylindrica* (L.) Raeusch.	Poaceae		Heat clearing, stopping bleeding, inducing diuresis	Decoction, external use	Root		5
*Lophatherum gracile* Brongn.	Poaceae		Heat clearing, relieving cough, inducing diuresis	Herbal tea	Root	JH-243	4
*Pennisetum alopecuroides* (L.) Spreng.	Poaceae		Heat clearing and detoxifying, relieving cough	Herbal tea	Whole plant	JH-106	3
*Saccharum spontaneum* L.	Poaceae	Si Mao Cao	Heat clearing and detoxifying, treating cold, relieving cough	Decoction	Rhizome, stem	JH-276	4
*Eomecon chionantha* Hance	Papaveraceae	Xue San Qi	Promoting blood circulation	Decoction	Root, rhizome	JH-219	2
*Macleaya cordata* (Willd.) R. Br.	Papaveraceae	Ye Xia Shuang	Skin disease	Herbal tea, medicinal bath	Whole plant	JH-253	3
*Akebia trifoliata* (Thunb.) Koidz.	Lardizabalaceae		Treating rheumatism, inducing diuresis, treating gynopathy, relaxing tendons and activating collaterals	Making tincture, medicinal bath	Root, stem, fruit	JH-296	6
*Sargentodoxa cuneata* (Oliv.) Rehder et E. H. Wilson	Lardizabalaceae	Huo Xue Feng	Treating gastrointestinal disease, heat clearing and detoxifying, promoting blood circulation, treating rheumatism	Making tincture, medicinal bath	Root, stem	JH-161	6
*Stephania cephalantha* Hayata	Menispermaceae	Sei Dong	Treating innominate inflammatory	Decoction	Tuber	JH-168	2
*Stephania kwangsiensis* H. S. Lo	Menispermaceae		Heat clearing and detoxifying, promoting blood circulation, relieving pain	Decoction	Tuber	JH-053	4
*Tinospora sagittata* (Oliv.) Gagnep.	Menispermaceae	Qing Teng	Heat clearing and detoxifying, diminishing inflammation, relieving pain, relieving sore throat	Decoction	Tuber	JH-231	5
*Berberis julianae* C. K. Schneid.	Berberidaceae		Heat clearing and detoxifying, diminishing inflammation, sterilization	Medicinal bath	Root	JH-247	4
*Dysosma versipellis* (Hance) M. Cheng	Berberidaceae		Heat clearing and detoxifying, promoting blood circulation	Decoction	Rhizome	JH-235	3
*Epimedium brevicornu* Maxim.	Berberidaceae		Nourishing, skin disease	Medicinal bath	Whole plant	JH-294	3
*Mahonia fortunei* (Lindl.) Fedde	Berberidaceae		Heat clearing and detoxifying	Decoction, medicinal bath	Root, stem	JH-241	3
*Nandina domestica* Thunb.	Berberidaceae		Heat clearing, treating rheumatism	Medicinal bath	Root, stem	JH-072	3
*Aconitum gymnandrum* Maxim.	Ranunculaceae		Treating rheumatism, traumatic injury	External use, medicinal bath	Whole plant	JH-163	4
*Clematis henryi* Oliv.	Ranunculaceae	Di Lei	Traumatic injury, reducing phlegm, relieving pain, relieving cough	Herbal tea, making tincture	Root, leaf	JH-026	6
*Clematis uncinata* Champ. ex Benth.	Ranunculaceae		Treating rheumatism, rheumatic arthritis, stopping bleeding, toothache, relaxing tendons and activating collaterals	Root: making tincture; decoction	Root, leaf	JH-155	7
*Liquidambar formosana* Hance	Altingiaceae	Lu Lu Tong	Relaxing tendons and activating collaterals	Medicinal bath	Fruit	JH-167	2
*Semiliquidambar cathayensis* H. T. Chang	Altingiaceae	Ban Feng He	Treating rheumatism, relaxing tendons and activating collaterals, promoting blood circulation, postpartum recovery, skin disease	Medicinal bath, decoction,	Bark, root	JH-284	7
*Loropetalum chinense* (R. Br.) Oliv.	Hamamelidaceae		Promoting blood circulation, leaf: stopping bleeding, traumatic injury	Medicinal bath, external use	Root, leaf	JH-103	5
*Astilbe rivularis* Buch.-Ham. ex D.Don	Saxifragaceae		Treating rheumatism, promoting blood circulation, relieving pain, treating gastrointestinal disease	Herbal tea	Rhizome	JH-032	5
*Hylotelephium erythrostictum* (Miq.) H. Ohba	Crassulaceae		Traumatic injury, treating innominate inflammatory, treating rheumatism	Medicinal bath	Whole plant	JH-126	4
*Kalanchoe pinnatum* (Lam.) Oken	Crassulaceae		Traumatic injury, treating innominate inflammation	Medicinal bath	Leaf	JH-300	3
*Sedum emarginatum* Migo	Crassulaceae		Heat clearing and detoxifying, traumatic injury, stopping bleeding, hepatitis	Decoction	Whole plant	JH-123	5
*Sedum kamtschaticum* Fisch.	Crassulaceae	Luo Di Sheng Gen	Treating innominate inflammation, traumatic injury, promoting blood circulation, stopping bleeding	Decoction	Whole plant	JH-242	5
*Ampelopsis grossedentata* (Hand.-Mazz.) W. T. Wang	Vitaceae	Tian Cha	Treating respiratory disease, heat clearing and detoxifying, treating hypertension	Herbal tea	Tender stem, leaf	JH-120	4
*Cayratia japonica* (Thunb.) Gagnep.	Vitaceae		Heat clearing and detoxifying, inducing diuresis, treating snake bite	Decoction, external use	Whole plant	JH-108	5
*Parthenocissus tricuspidata* (Siebold & Zucc.) Planch.	Vitaceae	Da Feng Teng	Treating rheumatism, promoting blood circulation	Herbal tea, medicinal bath	Root, stem, fruit	JH-266	4
*Bauhinia championii* (Benth.) Benth.	Fabaceae	Jiu Long Zuan	Treating rheumatism, relaxing tendons and activating collaterals, relieving pain	Herbal tea, medicinal bath	Stem	JH-285	5
*Callerya speciosa* (Champ. ex Benth.) Schot	Fabaceae	Tu Ren Shen	Nourishing, heat clearing, activating collaterals	Decoction	Root	JH-269	4
*Cassia tora* L.	Fabaceae		Improving eyesight, inducing diuresis, treating gastrointestinal disease	Food, medicinal bath	Seed	JH-240	5
*Desmodium multiflorum* DC.	Fabaceae	E Ma Huang	Heat clearing, treating infantile malnutrition	Herbal tea	Flower, branch	JH-144	3
*Entada phaseoloides* (L.) Merr.	Fabaceae	Niu Gu Feng	Treating rheumatism, nourishing, promoting blood circulation	Decoction	Rattan	JH-143	4
*Flemingia philippinensis* Merr. et Rolfe	Fabaceae	Diao Ma Zhuang	Nourishing	Decoction	Root	JH-012	2
*Gleditsia sinensis* Lam.	Fabaceae		Skin disease, eliminating phlegm, inducing diuresis, expelling parasite	Burnt, herbal tea, medicinal bath	Pod, seed, shoot thorn	JH-256	7
*Indigofera decora* Lindl. var. *ichangensis* (Craib) Y. Y. Fang & C. Z. Zheng	Fabaceae	Ye Jue Ming	Treating high fever	Herbal tea, medicinal bath	Root	JH-080	3
*Kummerowia striata* (Thunb.) Schindl.	Fabaceae	Hong Cha Zi	Heat clearing and detoxifying, promoting blood circulation, treating gastrointestinal disease	Medicinal bath, decoction	Whole plant	JH-290	5
*Lespedeza cuneata* (Dum. Cours.) G. Don	Fabaceae		Heat clearing and detoxifying, improving eyesight, treating infantile malnutrition	Herbal tea, medicinal bath	Whole plant	JH-292	5
*Millettia dielsiana* Harms	Fabaceae	Xing Xue Feng	Treating rheumatism, relaxing tendons and activating collaterals	Medicinal bath	Stem	JH-036	3
*Ohwia caudata* (Thunb.) H.Ohashi	Fabaceae		Heat clearing and detoxifying, treating rheumatism, skin disease	Medicinal bath	Root, whole plant	JH-274	4
*Pithecellobium clypearia* (Jack) Benth.	Fabaceae	Zao Ga Zi	Treating rheumatism, skin disease	Medicinal bath	Fruit	JH-110	3
*Sophora tonkinensis* Gagnep.	Fabaceae	Tao Ma Zhua	Heat clearing and detoxifying, diminishing inflammation, relieving pain,	Food (stewing with meat), medicinal bath	Root	JH-124	5
*Spatholobus suberectus* Dunn	Fabaceae	Jiu Ceng Feng	Promoting blood circulation, treating rheumatism	Food (stewing soup), medicinal bath	Stem	JH-054	4
*Fagopyrum acutatum* (Lehm.) Mansf. ex K. Hammer	Polygonaceae	Tie Leng Jiao	Heat clearing and detoxifying, promoting blood circulation, treating calculus	External use, decoction	Root, rhizome	JH-230	5
*Polygala fallax* Hemsl.	Polygalaceae	Huang Ji Gong	Nourishing	Food (stewing with chicken)	Root	JH-031	2
*Polygala japonica* Houtt.	Polygalaceae		Resolving phlegm, heat clearing and detoxifying	Herbal tea, stewing soup	Whole plant	JH-037	4
*Polygala tenuifolia* Willd.	Polygalaceae		Nourishing, resolving phlegm, strengthening muscles and bones	Decoction, medicinal bath	Bark	JH-191	5
*Polygonum hydropiper* L.	Polygonaceae	Liao Zi Cao	Treating rheumatism, detoxifying, expelling parasite, eliminating inflammation	Medicinal bath, making tincture	Whole plant	JH-199	6
*Polygonum perfoliatum* L.	Polygonaceae	She Bu Guo	Heat clearing and detoxifying, inducing diuresis, treating venomous snake bite	Medicinal bath	Whole plant	JH-084	4
*Reynoutria multiflora* (Thunb.) Moldenke	Polygonaceae		Nourishing	Decoction, medicinal bath	Tuber	JH-192	3
*Rumex acetosa* L.	Polygonaceae	Yang Ti Gen	Skin disease, heat clearing and detoxifying	Medicinal bath	Whole plant	JH-044	3
*Rumex nepalensis* Spreng.	Polygonaceae	Tu Da Huang	Relieving pain, stopping bleeding	Medicinal bath	Root, leaf	JH-218	3
*Agrimonia pilosa* Ledeb.	Rosaceae	Sa Yao	Treating gastrointestinal disease, diminishing inflammation, stopping bleeding, treating heatstroke	Medicinal bath, medicine, herbal tea	Whole plant	JH-098	7
*Geum aleppicum* Jacq.	Rosaceae		Treating rheumatism, heat clearing, relieving pain	Herbal tea, medicinal bath	Whole plant	JH-100	5
*Potentilla discolor* Bunge	Rosaceae		Heat clearing and detoxifying, stopping bleeding, treating diabetes	Decoction	Whole plant	JH-190	4
*Sanguisorba officinalis* L.	Rosaceae	Xi Gua Xiang	Heat clearing and detoxifying, stopping bleeding, relieving pain	Decoction, food (stewing with water)	Root	JH-209	5
*Frangula crenata* (Siebold & Zucc.) Miq.	Rhamnaceae		Heat clearing and detoxifying, expelling parasite	Decoction	Whole plant	JH-071	3
*Rhamnus globosa* Bunge	Rhamnaceae		Heat clearing and detoxifying, expelling parasite	Decoction	Fruit	JH-273	3
*Sageretia thea* (Osbeck) M. C. Johnst.	Rhamnaceae	Dao Ding Feng	Eliminating phlegm, skin disease, treating rheumatism	Decoction, medicinal bath	Aerial part	JH-198	5
*Zelkova serrata* (Thunb.) Makino	Ulmaceae	Sha Lang Shu	Treating gastrointestinal disease, skin disease	Medicinal bath	Bark, leaf	JH-014	3
*Humulus scandens* (Lour.) Merr.	Cannabaceae	Pi Jiu Hua	Heat clearing and detoxifying, inducing diuresis	Decoction, medicinal bath	Whole plant	JH-226	4
*Ficus pumila* L.	Moraceae	Hei Pi Feng	Nourishing, treating rheumatism	Herbal tea	Fruit	JH-002	3
*Boehmeria nivea* (L.) Gaudich.	Urticaceae		Heat clearing, inducing diuresis, stopping bleeding, nourishing	Medicinal bath, decoction	Rhizome, leaf	JH-291	6
*Parietaria micrantha* Ledeb.	Urticaceae	Shi Qian Cao	Heat clearing, promoting digest	Herbal tea	Whole plant	JH-099	3
*Pilea cavaleriei* H. Lév.	Urticaceae	Ai Jiao Cha	Relieving cough, detoxifying, heat clearing and detoxifying, relieving pain	Herbal tea	Whole plant	JH-194	3
*Hemsleya macrosperma* C.Y. Wu	Cucurbitaceae	Shan Wu Gui	Heat clearing and detoxifying, treating gastrointestinal disease	Decoction	Tuber	JH-283	3
*Thladiantha dubia* Bunge	Cucurbitaceae		Heat clearing and detoxifying, promoting blood circulation, relieving cough	Decoction	Fruit, root	JH-187	4
*Begonia cathayana* Hemsl.	Begoniaceae		Treating rheumatism, promoting blood circulation, skin disease, traumatic injury	Medicinal bath	Whole plant	JH-015	5
*Begonia fimbristipula* Hance	Begoniaceae	San Xue Zi	Treating traumatic injury, relieving cough	External use, decoction	Corm	JH-063	4
*Celastrus orbiculatus* Thunb.	Celastraceae		Heat clearing and detoxifying, treating rheumatism	Medicinal bath, decoction	Fruit	JH-287	4
*Celastrus wilfordii* Hook.f.	Celastraceae	Nan She Feng	Treating rheumatism	Medicinal bath, decoction	Whole plant	JH-118	3
*Euonymus fortunei* (Turcz.) Hand.-Mazz.	Celastraceae	Luo Shi Teng	Relaxing tendons and activating collaterals	Herbal tea, food (making soup)	Stem, leaf	JH-066	3
*Hypericum japonicum* Thunb.	Clusiaceae	Gua Zi Cao	Heat clearing and detoxifying promoting blood circulation, treating gastrointestinal disease	Decoction	Whole plant	JH-189	3
*Hypericum monogynum* L.	Clusiaceae		Treating rheumatism, relieving cough, treating stomachache, treating traumatic injury	Herbal tea	Root	JH-140	5
*Hypericum sampsonii* Hance	Clusiaceae		Treating gynopathy, heat clearing and detoxifying, relaxing tendons and activating collaterals	Herbal tea, medicinal bath	Whole plant	JH-131	5
*Viola inconspicua* Blume	Violaceae	Li Tou Cao	Heat clearing and detoxifying, promoting blood circulation, traumatic injury	Herbal tea	Whole plant	JH-252	4
*Croton congestus* Lour.	Salicaceae		Treating rheumatism	Medicinal bath, fruit: food	Branch, leaf	JH-013	3
*Bischofia polycarpa* (H. Lév.) Airy Shaw	Euphorbiaceae		Stopping bleeding	Medicinal bath	Root, bark	JH-087	2
*Glochidion puberum* (L.) Hutch.	Euphorbiaceae		Heat clearing and detoxifying, treating gastrointestinal disease, promoting blood circulation	Decoction	Root	JH-091	4
*Phyllanthus urinaria* L.	Phyllanthaceae	Ni Qiu Cao	Improving eyesight, heat clearing, promoting digest system	Decoction	Whole plant, root	JH-083	4
*Combretum indicum* (L.) DeFilipps	Combretaceae		Promoting digest, expelling parasite	Food	Seed	JH-224	3
*Lythrum salicaria* L.	Lythraceae	Hong Si Cao	Treating infantile malnutrition, stopping bleeding	Decoction	Whole plant	JH-148	3
*Rotala rotundifolia* (Buch.-Ham. ex Roxb.) Koehne	Lythraceae		Heat clearing, traumatic injury, treating snake bite, skin disease	Decoction, external use, medicinal bath	Whole plant	JH-272	7
*Melastoma dodecandrum* Lour.	Melastomataceae	Di Yang mei	Treating gastrointestinal disease	Decoction	Whole plant	JH-263	2
*Memecylon scutellatum* (Lour.) Hook. & Arn	Melastomataceae		Treating heart disease	Decoction	Flower	JH-157	2
*Osbeckia stellata* Buch.-Ham. ex Ker Gawl.	Melastomataceae		Diminishing inflammation, treating gastrointestinal disease, heat clearing, stopping bleeding	Decoction, food (stewing with meat)	Whole plant, root	JH-115	6
*Stachyurus chinensis* Franch.	Stachyuraceae		Treating gynopathy, heat clearing, urinary tract infection, inducing diuresis	Decoction	Stem pith	JH-068	5
*Acer pictum* Thunb.	Anacardiaceae		Treating rheumatism, traumatic injury	Decoction, external use, medicinal bath	Stem, leaf	JH-021	5
*Rhus chinensis* Mill.	Anacardiaceae	Pen Bai	Skin disease	Herbal tea, medicinal bath	Root, leaf	JH-258	3
*Acronychia pedunculata* (L.) Miq.	Rutaceae	La Jiang Ye	Detoxifying	Medicinal bath	Root, leaf, fruit	JH-052	2
*Atalantia buxifolia* (Poir.) Oliv.	Rutaceae	Lei Gong Le	Treating cold, treating rheumatism, treating respiratory disease, treating gastrointestinal disease, traumatic injury	Medicinal bath	Root, leaf	JH-067	6
*Citrus trifoliata* L.	Rutaceae		Skin disease	Medicinal bath	Branches and leaves	JH-171	2
*Toddalia asiatica* (L.) Lam.	Rutaceae	Zou Xue Feng	Treating rheumatism, relieving pain, promoting blood circulation	Medicinal bath	Root, leaf	JH-249	4
*Zanthoxylum ailanthoides* Siebold & Zucc.	Rutaceae		Treating rheumatism, relaxing tendons and activating collaterals	Making soup	Whole plant	JH-279	3
*Zanthoxylum armatum* DC.	Rutaceae		Treating rheumatism, relaxing tendons and activating collaterals	Food	Whole plant, fruit	JH-259	3
*Zanthoxylum austrosinense* Huang	Rutaceae	Man Shan Xiang	Treating rheumatism, promoting blood circulation	Medicinal bath, external use, decoction	Fruit	JH-304	5
*Melia azedarach* L.	Meliaceae		Expelling parasite, skin disease	Decoction, external use	Root, bark	JH-232	4
*Sida acuta* Burm.f.	Malvaceae		Diminishing inflammation, sterilization	Medicinal bath	Root, leaf	JH-207	3
*Wikstroemia indica* (L.) C. A. Mey.	Thymelaeaceae	Tie Gu Shan	Skin disease	Medicinal bath	Whole plant	JH-129	2
*Rorippa indica* (L.) Hiern	Brassicaceae	Mi Gong	Stopping bleeding, traumatic injury, relieving cough, skin disease	Herbal tea, medicinal bath	Whole plant	JH-092	6
*Balanophora harlandii* Hook.f.	Balanophoraceae		Traumatic injury, promoting blood circulation, treating gynopathy disease	Medicinal bath	Whole plant	JH-132	4
*Taxillus chinensis* (DC.) Danser	Loranthaceae		Treating rheumatism, nourishing, strengthening muscles and bones, miscarriage prevention	Medicinal bath	Whole plant	JH-079	5
*Viscum articulatum* Burm.f.	Loranthaceae	Pang Xie Jiao	Treating rheumatism, treating respiratory disease, promoting blood circulation	Herbal tea, medicinal bath	Branch, leaf	JH-211	5
*Viscum diospyrosicola* Hayata	Loranthaceae	Tao Ji Sheng	Treating rheumatism, heat clearing, diminishing inflammation, relaxing tendons	Decoction, medicinal bath	Whole plant	JH-111	6
*Viscum liquidambaricola* Hayata	Loranthaceae		Treating rheumatism, relaxing tendons and activating collaterals, promoting blood circulation, relieving cough	Decoction, medicinal bath	Branch, leaf	JH-107	6
*Ceratostigma willmottianum* Stapf	Plumbaginaceae		Treating gynopathy, treating rheumatism, treating respiratory disease	Medicinal bath	Branches and leaves, root	JH-261	4
*Plumbago zeylanica* L.	Plumbaginaceae	Bai Zi Cao	Treating rheumatism, promoting blood circulation, expelling parasite, detoxifying	External use, making tincture	Whole plant, root	JH-065	6
*Plumbago zeylanica* L.	Plumbaginaceae	Meng Lao Hu	Treating rheumatism, detoxifying, promoting blood circulation, skin disease	Medicinal bath	Root, leaf	JH-175	4
*Drosera peltata* Thunb.	Droseraceae	Di Ming Zhu	Traumatic injury, detoxifying	Medicinal bath	Whole plant	JH-064	3
*Achyranthes aspera* L.	Amaranthaceae	Bai Niu Xi	Heat clearing and detoxifying, treating rheumatism, nourishing, relieving pain	Decoction, medicinal bath	Whole plant	JH-267	6
*Achyranthes bidentata* Blume	Amaranthaceae	Tu Niu Xi	Nourishing	Decoction	Root	JH-050	2
*Achyranthes longifolia* (Makino) Makino	Amaranthaceae	Hong Niu Xi	Promoting blood circulation, inducing diuresis	Food	Root	JH-227	3
*Aerva sanguinolenta* (L.) Blume	Amaranthaceae		Relieving cough, traumatic injury, strengthening muscles and bones, treating dysentery, nourishing	Medicinal bath, food	Root, flower	JH-078	7
*Amaranthus spinosus* L.	Amaranthaceae		Heat clearing and detoxifying	Medicinal bath	Whole plant	JH-200	2
*Phytolacca acinosa* Roxb.	Phytolaccaceae		Traumatic injury, skin disease	Root: external use; tender leaf and stem: food	Root	JH-112	4
*Basella alba* L.	Basellaceae	Teng Sa Qi	Heat clearing and detoxifying, skin disease	Decoction, external use	Leaf, whole plant	JH-119	4
*Portulaca oleracea* L.	Portulacaceae	Gua Zi Cai	Heat clearing and detoxifying, eliminating phlegm	Medicinal bath, herbal tea	Whole plant	JH-007	4
*Talinum paniculatum* (Jacq.) Gaertn.	Portulacaceae	Tu Ren Shen	Nourishing, inducing saliva, detoxifying	Food	Tuber	JH-301	4
*Ardisia affinis* Hemsl.	Primulaceae	Xiao Ai Di Cha	Promoting blood circulation, traumatic injury	Decoction, medicinal bath	Root	JH-095	4
*Ardisia corymbifera* Mez	Primulaceae		Traumatic injury, treating rheumatism	Medicinal bath	Whole plant	JH-028	3
*Ardisia crenata* Sims var. *bicolor* (E. Walker) C. Y. Wu & C. Chen	Primulaceae	Zhen Zhu Gai Liang San	Treating traumatic injury, treating rheumatism, treating respiratory disease	Food, medicinal bath, herbal tea	Whole plant	JH-254	6
*Ardisia cymosa* Blume	Primulaceae		Promoting blood circulation, heat clearing, diminishing inflammation, stopping bleeding	Decoction	Whole plant	JH-001	5
*Ardisia gigantifolia* Stapf	Primulaceae		Treating rheumatism, promoting blood circulation, relieving pain	External use, medicinal bath	Rhizome, whole plant	JH-170	5
*Ardisia japonica* (Thunb.) Blume	Primulaceae	Xue Feng	Treating rheumatism, promoting blood circulation, skin disease, treating cold, relieving cough	Herbal tea, medicinal bath	Whole plant, root	JH-121	7
*Ardisia pusilla* A. DC.	Primulaceae		Relieving pain, promoting blood circulation, treating gynopathy, treating snake bite, skin disease	Medicinal bath	Whole plant	JH-225	6
*Embelia laeta* (L.) Mez	Primulaceae	Zhuan Guo Hong	Treating rheumatism	Medicinal bath, stewing soup	Whole plant	JH-048	3
*Embelia rudis* Hand.-Mazz.	Primulaceae	Gou She Feng	Treating rheumatism, skin disease	Herbal tea, medicinal bath	Whole plant	JH-004	4
*Lysimachia barystachys* Bunge	Primulaceae		Skin disease, stopping bleeding	Medicinal bath, external use	Whole plant	JH-210	4
*Plantago asiatica* L.	Primulaceae	Ma Guai Cao	Heat clearing and detoxifying, inducing diuresis, eliminating phlegm	Herbal tea	Whole plant	JH-018	4
*Camellia sinensis* (L.) Kuntze	Theaceae		Heat clearing, inducing diuresis, relieving cough, treating heatstroke	Medicinal bath, herbal tea	Tender leaf	JH-020	6
*Symplocos paniculata* Miq.	Symplocaceae		Heat clearing, treating rheumatism	Medicinal bath, decoction	Stem and leaf	JH-305	4
*Gaultheria leucocarpa* var. *yunnanensis* (Franch.) T. Z. Hsu & R. C. Fang	Ericaceae	Xia Shan Hu	Treating rheumatism, promoting blood circulation, relaxing tendons and activating collaterals	Medicinal bath	Whole plant	JH-082	4
*Eucommia ulmoides* Oliv.	Eucommiaceae		Nourishing, strengthening muscles and bones, miscarriage prevention	Medicinal bath	Bark	JH-205	4
*Cephalanthus subspinosns* (Roxb.) Ridsd. et Bakh. f.	Rubiaceae		Skin disease	Herbal tea, medicinal bath	Whole plant	JH-125	3
*Damnacanthus giganteus* (Makino) Nakai	Rubiaceae	Xiu Hua Zhen	Nourishing, stopping bleeding	Herbal tea, decoction	Whole plant	JH-302	4
*Damnacanthus indicus* C. F. Gaertn.	Rubiaceae	Xiu Hua Zhen	Treating infantile malnutrition, nourishing, relieving pain, treating cold, treating hepatitis	Herbal tea, food (making soup)	Whole plant	JH-234	7
*Hedyotis auricularia* L.	Rubiaceae	Huang Shao	Heat clearing and detoxifying, treating gastrointestinal disease, relieving cough, treating cold, promoting blood circulation, skin disease, snake bite	Herbal tea, medicinal bath	Leaf	JH-206	9
*Paederia scandens* (Lour.) Merr.	Rubiaceae	Ji Shi Teng	Treating rheumatism, promoting digest, heat clearing and detoxifying	Medicinal bath, herbal tea, decoction	Whole plant	JH-074	6
*Serissa serissoides* (DC.) Druce	Rubiaceae		Treating rheumatism, heat clearing and detoxifying, relaxing tendons and activating collaterals	Decoction, medicinal bath	Whole plant	JH-051	5
*Uncaria rhynchophylla* (Miq.) Miq. ex Havil.	Rubiaceae	Ying Zhao Feng	Treating rheumatism, promoting blood circulation	Medicinal bath	Branch, leaf	JH-038	3
*Adenium obesum* (Forssk.) Roem.& Schult.	Apocynaceae		Treating gastrointestinal disease, treating gynopathy	External use	Flower	JH-268	3
*Anodendron affine* (Hook. & Arn.) Druce	Apocynaceae		Treating rheumatism	Medicinal bath	Whole plant	JH-158	2
*Cynanchum auriculatum* Royle ex Wight	Apocynaceae	Niu Pi Dong	Skin disease	Herbal tea, medicinal bath	Whole plant	JH-176	3
*Cynanchum paniculatum* (Bunge) Kitag.	Apocynaceae	Xu Chang Qin	Heat clearing, diminishing inflammation, relieving cough	Herbal tea, medicinal bath	Whole plant	JH-278	5
*Dischidia australis* Tsiang et P. T. Li	Apocynaceae		Treating respiratory disease, skin disease, diminishing inflammation, treating arthritis	Herbal tea	Whole plant	JH-127	5
*Dischidia chinensis* Champ. ex Benth.	Apocynaceae	Shi Xin Zi	Heat clearing and detoxifying, reducing phlegm, treating infantile malnutrition	Food (stewing with meat)	Whole plant	JH-139	4
*Marsdenia sinensis* Hemsl.	Apocynaceae	Jiu Niu Teng	Treating rheumatism, promoting blood circulation, treating heatstroke	Decoction	Stem	JH-151	4
*Trachelospermum jasminoides* (Lindl.) Lem.	Apocynaceae	Guo Qiang Feng	Treating rheumatism	Decoction, medicinal bath	Whole plant	JH-045	3
*Argyreia acuta* Lour.	Convolvulaceae		Skin disease	Medicinal bath	Whole plant	JH-177	2
*Cuscuta chinensis* Lam.	Convolvulaceae		Nourishing	Herbal tea, food	Seed	JH-286	3
*Dichondra repens* J. R. Forst. & G. Forst.	Convolvulaceae		Heat clearing and detoxifying, expelling parasite	Decoction	Whole plant	JH-270	3
*Petrocodon dealbatus* var. *dealbatus*	Gesneriaceae	Bei Feng Fei Yang	Relieving cough	Decoction	Whole plant	JH-237	2
*Buddleja lindleyana* Fortune	Scrophulariaceae	Yang Wei Ba	Skin disease, treating skin itch	Medicinal bath	Whole plant	JH-086	3
*Andrographis paniculata* (Burm.f.) Nees	Acanthaceae		Heat clearing and detoxifying, eliminating inflammation	Herbal tea	Whole plant	JH-141	3
*Campsis grandiflora* (Thunb.) K. Schum.	Bignoniaceae	Hong Hua Dao Shui Lian	Traumatic injury	Herbal tea, medicinal bath	Whole plant	JH-034	3
*Radermachera sinica* (Hance) Hemsl.	Bignoniaceae		Heat clearing, treating venomous snake bite, sterilization	External use (leaf), medicinal bath	Root, leaf, fruit, branch	JH-009	5
*Callicarpa pedunculata* R. Br.	Verbenaceae		Skin disease	Medicinal bath	Stem, leaf	JH-160	2
*Clerodendrum chinense* (Osbeck) Mabb.	Verbenaceae		Treating rheumatism, promoting blood circulation, relieving pain, heat clearing and detoxifying, improving digestion	Herbal tea	Root, leaf, whole plant	JH-164	6
*Verbena officinalis* L.	Verbenaceae	Tie Ma Bian	Treating rheumatism, treating venomous snake bite, heat clearing, promoting blood circulation, eliminating inflammation	Decoction, external use, medicinal bath	Whole plant	JH-135	8
*Vitex negundo* L.	Verbenaceae	Huang Jin Zi	Nourishing, relieving cough, reducing phlegm	Medicinal bath, food (stewing with meat)	Whole plant	JH-248	5
*Clerodendrum cyrtophyllum* Turcz.	Lamiaceae		Heat clearing and detoxifying, treating rheumatism	Decoction, medicinal bath	Root, leaf	JH-142	4
*Leonurus japonicus* Houtt.	Lamiaceae	Hong Hua Ai	Heat clearing	Herbal tea, medicinal bath, making soup	Whole plant	JH-075	3
*Lycopus lucidus* Turcz. ex Benth.	Lamiaceae		Treating rheumatism	Decoction	Whole plant	JH-033	2
*Mentha canadensis* L.	Lamiaceae		Treating cold, skin disease	Food (stewing with meat), medicinal bath	Whole plant	JH-117	4
*Mosla chinensis* Maxim.	Lamiaceae	Xiao Ye Suo Cao	Preventing heatstroke, mosquitoes repelling	Herbal tea, medicinal bath	Whole plant	JH-019	4
*Perilla frutescens* (L.) Britton	Lamiaceae		Detoxifying, treating respiratory disease, treating cold, invigorating stomach	Medicinal bath, food (stir-fry)	Stem, leaf, fruit	JH-023	5
*Pogostemon auricularius* (L.) Hassk.	Lamiaceae	Ye ji wei	Heat clearing, cleaning the wound	Decoction	Whole plant	JH-239	3
*Prunella vulgaris* L.	Lamiaceae		Improving eyesight, promoting blood circulation	Herbal tea	Fruit cluster, flower	JH-179	3
*Scutellaria barbata* D. Don	Lamiaceae		Heat clearing and detoxifying, inducing diuresis, treating cold	Decoction	Whole plant	JH-042	3
*Stachys geobombycis* C.Y. Wu	Lamiaceae		Detoxifying, treating gastrointestinal disease, traumatic injury, skin disease	Food	Whole plant, rhizome	JH-228	4
*Codonopsis javanica* (Blume) Hook.f. & Thomson	Campanulaceae	Nai Shen	Treating gastrointestinal disease, nourishing, relieving cough, treating gynopathy, treating infantile malnutrition	Food (stewing with meat)	Root	JH-154	6
*Codonopsis lanceolata* (Siebold & Zucc.) Benth. & Hook.f. ex Trautv.	Campanulaceae	Yang Ru	Tonic	Food (cooking with meat)			2
*Ilex asprella* (Hook. & Arn.) Champ. ex Benth. var. *asprella*	Aquifoliaceae	Cheng Xing Shu	Promoting blood circulation, clearing heat	Herbal tea, decoction, medicinal bath	Leaf, root	JH-303	5
*Ilex chinensis* Sims	Aquifoliaceae		Sterilization, promoting blood circulation	Leaf: medicinal bath; seed: making tincture, decoction; bark: decoction	Bark, leaf, root, seed	JH-182	6
*Achillea millefolium* L.	Asteraceae	Suan Ming Cao	Treating rheumatism, traumatic injury, treating gynopathy, snake bite	External use, decoction, medicinal bath	Leaf, flower	JH-016	7
*Ageratum conyzoides* L.	Asteraceae	Bai Hua Cao	Heat clearing and detoxifying, diminishing inflammation, stopping bleeding	External use	Whole plant	JH-257	4
*Artemisia annua* L.	Asteraceae	Qing Hao	Treating malaria, skin disease	Medicinal bath	Branches and leaves	JH-238	3
*Artemisia argyi* H. Lév. & Vaniot	Asteraceae	Ye Ai	Skin disease, treating gynopathy	Herbal tea, medicinal bath	Whole plant	JH-005	4
*Artemisia capillaris* Thunb.	Asteraceae		Treating gastrointestinal disease, diminishing inflammation	Medicinal bath, decoction	Tender shoot, tender leaf	JH-062	4
*Artemisia dubia* Wall. ex Bess.	Asteraceae		Treating rheumatism, heat clearing and detoxifying, diminishing inflammation, expelling parasite	Decoction, external use, medicinal bath	Whole plant	JH-156	7
*Artemisia princeps* Pamp.	Asteraceae		Treating rheumatism, nourishing, treating gynopathy, diminishing inflammation, stopping bleeding	Decoction	Leaf	JH-245	6
*Aster indicus var. indicus* (L.) Sch.-Bip.	Asteraceae	Ji You Cai	Heat clearing, relieving cough	Herbal tea, medicinal bath	Whole plant	JH-188	4
*Aster tataricus* L.f.	Asteraceae	Ji You Cha	Heat clearing	Herbal tea	Root	JH-003	2
*Centipeda minima* (L.) A. Braun & Asch.	Asteraceae	E Bu Shi Cao	Treating rheumatism, promoting blood circulation, eliminating inflammation	Decoction, external use, medicinal bath	Whole plant	JH-162	6
*Cirsium japonicum* (Thunb.) Fisch. ex DC.	Asteraceae	Shan Luo Bo	Nourishing, treating gynopathy, promoting blood circulation, stopping bleeding, eliminating inflammation	Decoction, external use, medicinal bath	Whole plant, root	JH-215	8
*Eupatorium chinense* L.	Asteraceae		Treating rheumatism	Medicinal bath	Whole plant	JH-150	2
*Farfugium japonicum* (L.) Kitam.	Asteraceae		Treating gynopathy, traumatic injury, relieving cough	Decoction, external use, medicinal bath	Root	JH-280	6
*Gerbera anandria* (L.) Sch.-Bip.	Asteraceae	Pu Di Ling	Treating hepatitis	Decoction,	Whole plant	JH-255	2
*Gerbera piloselloides* (L.) Cass.	Asteraceae	Pu Di Gen	Heat clearing, diminishing inflammation, treating infantile malnutrition	Decoction, medicinal bath	Whole plant	JH-223	5
*Glebionis lavandulifolium* (Fisch. ex Trautv.) Ling & Shih	Asteraceae		Heat clearing and detoxifying	Herbal tea, medicinal bath	Whole plant	JH-166	3
*Glebionis morifolium* (Ramat.) Tznel.	Asteraceae		Heat clearing and detoxifying, treating rheumatism, improving eyesight	Herbal tea, medicinal bath	Flower	JH-047	5
*Grangea maderaspatana* (L.) Poir.	Asteraceae	Gua Zi Cao	Heat clearing, treating incised wound	External use, herbal tea	Whole plant	JH-201	4
*Gynura japonica* (Thunb.) Juel	Asteraceae		Treating diabetes, treating infantile malnutrition, traumatic injury	Decoction	Whole plant	JH-137	4
*Helianthus annuus* L.	Asteraceae		Treating rheumatism	Medicinal bath	Flower	JH-152	2
*Inula cappa* (Buch.-Ham. ex D.Don) DC.	Asteraceae	Bai Mian Feng	Treating rheumatism, relieving pain, relieving cough, treating cold, eliminating phlegm	Medicinal bath	Whole plant	JH-169	6
*Inula japonica* Thunb.	Asteraceae		Treating infantile malnutrition	Decoction	Root, leaf, flower	JH-172	2
*Senecio scandens* Buch.-Ham. ex D. Don	Asteraceae	Jiu Li Guang	Skin disease, improving eyesight, heat clearing and detoxifying	Herbal tea, medicinal bath	Whole plant	JH-076	5
*Viburnum odoratissimum* Ker Gawl.	Adoxaceae	Jian Gu Feng	Treating rheumatic arthritis, traumatic injury	Herbal tea, food, medicinal bath	Whole plant	JH-035	4
*Lonicera acuminata* Wall.	Caprifoliaceae	Yin hua	Skin disease	Medicinal bath	Whole plant	JH-186	2
*Lonicera confusa* (Sweet) DC.	Caprifoliaceae		Heat clearing and detoxifying	Decoction	Flower, stem, leaf	JH-149	2
*Lonicera hypoglauca* Miq.	Caprifoliaceae		Heat clearing and detoxifying, promoting blood circulation	Medicinal bath	Flower bud, stem	JH-022	3
*Lonicera japonica* Thunb.	Caprifoliaceae		Heat clearing and detoxifying, promoting blood circulation	Herbal tea, medicinal bath	Stem	JH-085	4
*Lonicera reticulata* Champ.	Caprifoliaceae	Yin hua	Skin disease	Medicinal bath	Whole plant	JH-104	2
*Pittosporum glabratum* Lindl.	Pittosporaceae	Tie Liang San	Treating steaming bone	Herbal tea, medicinal bath	Seed, skin	JH-173	3
*Dendropanax dentigerus* (Harms) Merr.	Araliaceae	Yin Yang Feng	Heat clearing and detoxifying, treating rheumatism, skin disease, relieving pain	Medicinal bath	Root, bark	JH-293	5
*Eleutherococcus nodiflorus* (Dunn) S. Y. Hu	Araliaceae	Wu Gu Gou	Nourishing	Food (stewing with chicken and soybean)	Root	JH-145	2
*Eleutherococcus senticosus* (Rupr. & Maxim.) Maxim.	Araliaceae	Wu Jia Pi	Nourishing	Medicinal bath	Leaf, root bark, stem	JH-288	2
*Gamblea ciliata* var. *evodiifolia* (Franch.) C. B. Shang, Lowry & Frodin	Araliaceae	Wu Zhao Feng	Treating rheumatism	Decoct, medicinal bath	Rhizome	JH-102	3
*Hedera sinensis* (Tobler) Hand.-Mazz.	Araliaceae	San Jiao Feng	Heat clearing and detoxifying, treating rheumatism, nourishing, relieving pain	Medicinal bath	Whole plant	JH-010	5
*Heteropanax fragrans* (Roxb.) Seem.	Araliaceae	Ya Jiao Feng	Treating rheumatism	Herbal tea, food (making soup)	Bark, stem pith	JH-220	3
*Kalopanax septemlobus* (Thunb.) Koidz	Araliaceae	Shan Ku Di Feng	Treating rheumatism, promoting blood circulation, relieving pain, traumatic injury	Medicinal bath	Bark, stem	JH-094	5
*Panax japonicus* (T. Nees) C. A. Mey.	Araliaceae		Nourishing, eliminating phlegm, stopping bleeding, relieving pain	Decoction	Rhizome	JH-244	5
*Schefflera heptaphylla* (L.) Frodin	Araliaceae	Ya Jiao Feng	Heat clearing and detoxifying, treating rheumatism, skin disease, relaxing tendons and activating collaterals	Decoction	Leaf, bark	JH-081	5
*Bupleurum chinense* DC.	Apiaceae	Tu Chai Hu	Diminishing inflammation, heat clearing, treating cold, treating fever	Herbal tea	Root	JH-030	5
*Cryptotaenia japonica* Hassk.	Apiaceae	Shui Qin Cai	Promoting blood circulation, skin disease, treating respiratory disease	Food, medicinal bath	Whole plant	JH-203	5
*Hydrocotyle sibthorpioides* Lam.	Apiaceae		Heat clearing, promoting digest, treating infantile malnutrition	Food, herbal tea, making soup	Whole plant	JH-060	6
*Peucedanum guangxiense* R. H. Shan & M. L. Sheh	Apiaceae		Treating cold, treating rheumatism	Decoction, medicinal bath	Root	JH-024	4
*Sanicula chinensis* Bunge	Apiaceae	Shan Qin Cai	Relieving cough, treating gastrointestinal disease, heat clearing, diminishing inflammation	Herbal tea	Whole plant	JH-025	5
*Pinus massoniana* Lamb.	Pinaceae		Treating rheumatism, relaxing tendons and activating collaterals	Medicinal bath	Branches and leaves	JH-212	3
Pteridophyta							
*Huperzia serrata* (Thunb.) Trevis	Huperziaceae	Qian Ceng Ta	Promoting blood circulation, stopping bleeding, relieving pain, treating senile dementia, traumatic injury	External use	Whole plant	JH-049	6
*Diphasiastrum complanatum* (L.) Holub	Lycopodiaceae	Song Jin Cao	Traumatic injury, treating rheumatism	Medicinal bath, decoction	Whole plant	JH-297	4
*Lycopodium japonicum* Thunb.	Lycopodiaceae	Sheng Jin Cao	Treating rheumatism, relaxing tendons and activating collaterals	Medicinal bath	Whole plant	JH-136	3
*Phlegmariurus fargesii* (Herter) Ching	Lycopodiaceae		Traumatic injury, treating rheumatism	Medicinal bath	Whole plant	JH-039	3
*Equisetum arvense* L.	Equisetaceae	Jie Jie Cao	Stopping bleeding	Decoction, external use	Whole plant	JH-289	3
*Equisetum ramosissimum subsp. debile* (Roxb. ex Vaucher) Hauke	Equisetaceae		Improving eyesight, inducing diuresis	Decoction, medicinal bath	Whole plant	JH-197	4
*Angiopteris fokiensis* Hieron.	Angiopteridaceae	Xiao Ma Ti	Heat clearing and detoxifying, promoting blood circulation, relieving pain	Decoction, external use	Rhizome	JH-222	5
*Lygodium japonicum* (Thunb.) Sw.	Lygodiaceae	Tie Xian Cao	Inducing diuresis, treating calculus, treating rheumatism	Medicinal bath; decoction	Spore, whole plant	JH-216	5
*Lygodium scandens* (L.) Sw.	Lygodiaceae		Heat clearing, inducing diuresis, relieving pain	Spore: decoction; medicinal bath	Spore, whole plant	JH-204	6
*Cibotium barometz* (L.) J. Sm.	Dicksoniaceae	Jin Gou Zi	Stopping bleeding	External use	Hair	JH-299	2
*Alsophila spinulosa* (Wall. ex Hook.) Tryon	Cyatheaceae	Long Gu Feng	Treating rheumatism, promoting blood circulation, strengthening muscles and bones	Medicinal bath	Stem	JH-229	4
*Pteris multifida* Poir.	Pteridaceae	Feng Wei Cao	Heat clearing and detoxifying, traumatic injury, treating gastrointestinal disease	Root: external use; food; medicinal bath	Whole plant	JH-008	6
*Aleuritopteris argentea* (Gmel.) Fée	Sinopteridaceae	Huo Shao Cao	Treating gynopathy, nourishing, treating empyrosis	External use	Whole plant	JH-250	4
*Davallia mariesii* T. Moore ex Baker	Davalliaceae		Traumatic injury	External use	Rhizome	JH-073	2
*Lepidogrammitis drymoglossoides* (Baker) Ching	Polypodiaceae	Pa Shan Hu	Heat clearing and detoxifying, inducing diuresis, stopping bleeding	Decoction	Whole plant	JH-057	4
*Lepidogrammitis rostrata* (Bedd.) Ching	Polypodiaceae	Bao Shu Lian	Treating infantile malnutrition, promoting digest	Making soup	Whole plant	JH-105	3
*Lepisorus thunbergianus* (Kaulf.) Ching	Polypodiaceae		Heat clearing, inducing diuresis, relieving cough	Decoction	Whole plant	JH-116	4
*Microsorum fortunei* (T. Moore) Ching	Polypodiaceae	Qi Xing Jian	Treating rheumatism	Herbal tea, medicinal bath	Whole plant	JH-059	3
*Pyrrosia lingua* (Thunb.) Farw.	Polypodiaceae		Traumatic injury	Medicinal bath, external use	Leaf	JH-056	3
*Pseudodrynaria coronans* (Wall. ex Mett.) Ching	Drynariaceae	Bi Shan Hu	Treating rheumatism, nourishing, relaxing tendons and activating collaterals, traumatic injury	Decoction, medicinal bath	Rhizome	JH-183	6
Lichenes							
*Usnea diffracta* Vain	Usneaceae	Song Jin Teng	Treating rheumatism	Herbal tea, food, medicinal bath	Whole plant	JH-147	4

The order of plant species in this table is followed by the APG IV system, gymnosperms classification system (1978), and Qinrenchang fern plant classification system (1978)

## Results and discussion

### Diversity and characteristics of medicinal plants

By conducting field surveys at the Dragon Boat Festival marketplace in Jianghua in 2016 and 2017, 306 species belonging to 249 genera and 113 families were recorded and identified (Table 1). The taxonomic statistics clearly demonstrate the plant species biodiversity present in this Yao community market. The plant family with the most species represented was Asteraceae (23 species). Fabaceae (Leguminosae) was the second most common plant family with 15 species while Primulaceae and Lamiaceae were the third and fourth largest plant families with 11 and 10 species, respectively. Regarding plant genera, most of genera had three or fewer species represented, except for the genera *Artemisia* and *Ardisia* (Table 1). Seven species of *Ardisia* were found in the marketplace, while five species of *Artemisia* were present. The genus *Ardisia*, which contains a large number of medicinal species, has more than a 900-year history of clinical use in China. Some *Ardisia* species are common ingredients of traditional Chinese medicine formulas and Chinese folk medicines, including all the *Ardisia* species identified in the Jianghua medicinal market.

Compared to the previous study by Liu [15] 15 years ago, the plant species number recorded in the current study has almost tripled, which indicates that the medicinal market in Jianghua has grown considerably. This change seems to be in paradox to the loss of traditional knowledge under the impact of rapid economic development. One of the reasons for the increase in plant diversity in the marketplace might be the improved transportation and living conditions in remote areas, which makes collection easier and helps to facilitate communication among different ethnic people and thus enhances the marketplace experience. On the contrary, elder informants (> 50 years old) could provide Yao names (Table 1) to only 173 plant species (56%). The local people used Mandarin Chinese instead of the Yao language to identify many of the medicinal plants in this survey. This phenomenon might partially reflect the gradual disappearance of the local medicine-associated knowledge. It could also be the result of merging of different medicinal culture from different groups of people. The Yao language, as a spoken language without traditional characters, can only be memorized and transmitted by humans; this might also explain the loss of local Yao language which leads to the lack of Yao names of medicinal plants.

### Plant parts used as medicine

The statistics of using parts of medicinal plants traded in the market are summarized (Table 2). Using whole plants is the most frequent method with 140 species, while using roots is the second one with 67 species. Using plant leaves (48 species) and stems (33 species) are less common. Normally, the local people traded leafed branches to use in medicinal baths according to our observations. The local people prefer to use fresh medicinal plants, and thus, the aerial parts of the plants were more abundant than roots in the marketplace.

**Table 2:** The used parts of medicinal plants traded in the market in Jianghua

Plant part	Records	Percentage	Plant part	Records	Percentage
Root	67	21.9	Fruit	18	5.9
Stem (branches)	33	10.8	Seed	6	2.0
Leaf	48	15.7	Rhizome	27	8.8
Stem pith	3	1.0	Bark	14	4.6
Flower	14	4.6	Whole plant	140	45.8

Regarding the plant parts used with their modality categories, (1) medicinal baths are the most common modality used by the Yao people which mostly use the leaves and the branches; (2) the reasons for using root, fruit, and flowers were quite diverse, including almost all modality categories; (3) most of the rhizomes were used for medicine, taking medicinal baths, or making herbal teas.

Most of the seeds from six species in total are edible. For example, the seeds of *Ilex chinensis* can be used for brewing. The seeds of some species like *Cuscuta chinensis*, *Gleditsia sinensis*, and *Pittosporum glabratum* can be used to make tea. The seeds of *Combretum indicum*, *Cuscuta chinensis*, and *Senna tora* can be cooked with other ingredients into a dish.

### Medicinal uses of plants

The medicinal uses of plants traded in the market are also various, with 27 types (Table 1). The top ten therapeutic medicinal uses are listed in Table 3. These ten medicinal uses reflect the most frequent physical ailments closely attributed to local climate, environment, and the type of work [20].

**Table 3:** The top ten medicinal uses of medicinal plants in the Yao marketplace in Jianghua

Medicinal uses	Records	Percentage	Medicinal uses	Records	Percentage
Treating rheumatism	106	34.6	Nourishing	45	14.7
Clearing heat	103	33.7	Treating traumatic injury	39	12.8
Detoxification	82	26.8	Relieving pain	33	10.8
Promoting blood circulation	57	18.6	Relieving cough	33	10.8
Treating skin diseases	45	14.7	Stopping bleeding	26	8.5

Most local Yao people living in humid and highland areas are engaged in heavy physical work for a living throughout the year [15], and thus, it is not surprising that rheumatism is the number one disorder in local communities. Remarkably, almost one third of the species (106) can be used to treat rheumatism. The cold and skin diseases are also common ailments in such an environment. Herbal medicine for skin diseases and relieving cough are important and frequently used. According to traditional Yao medicinal theory, a cold and humid environment will cause the closure of pores. The heat inside the human body cannot be excreted out on time, and thus, the balance of yin and yang will be broken and cause sickness. In order to solve this problem, local people use many different herbs to clear inner heat (33.7%) or detoxification (26.8%, relieving internal heat or fever). Moreover, it is much easier to have injuries when doing heavy physical work in mountainous environment. Herbal medicinal plants for treating traumatic injury, relieving pain, and stopping bleeding comprise a large part of the medicinal market. Herbs for nourishing and promoting blood circulation also comprise a large part of the market because they can effectively help local people to recover from injuries.

Yao medicine is renowned for being good at treating rheumatism and gynecological diseases [21]. One of our former studies found that red-headed Yao women like to use herbs like *Aeschynanthus bracteatus*, *Celosia argentea*, and *Sabia fasciculata* to make decoctions for medicinal baths so that they can return to farming work as soon as a week after giving birth [22]. Those herbs are believed to have very good anti-inflammatory and tonic effectiveness by local people. In the present study, no medicinal plant was mentioned for postpartum recovery or gynecological diseases by local people. Most of the herbs for nourishment or pain relief like *Amomum villosum*, *Anemarrhena asphodeloides*, *Vitex negundo*, and *Saururus chinensis* are regarded to be good for women according to local people.

### Modalities of medicinal plants

Eight categories of modalities of medicinal plants about the market were recorded. About 60% of plant species were used for medicinal baths, making it the most common traditional medicinal modality. Medicinal baths are a characteristic custom for the Yao ethnic group. When having a medicinal bath, the skin, as the largest human organ, can be fully exposed to the medicinal bath water so that certain medicinally useful molecules can be absorbed that way [23, 24]. The heat of the water can also stimulate the blood capillaries and lymph vessels to expand and promote blood circulation and metabolism [23, 24]. There are many aromatic plants used in medicinal baths like *Gaultheria leucocarpa* var. *yunnanensis*. The heat of the bath water can accelerate the volatile molecules to evaporate from medicinal plants, which can be absorbed by breathing and also strengthen the effectiveness of medicine [4].

Based on our investigation, one or more species of medicinal plants are typically immersed in hot water for bathing. The Yao people do not have settled formulas and precise amounts of medicinal plants for these baths. They usually put the plants with similar pharmacological efficacy together to enhance their effects. These Yao formulas have not been well studied scientifically, and side effects are not well documented. Therefore, further phytochemical, pharmacological, and clinical tests are needed to determine the safety and efficacy of these traditional practices [4].

Besides medicinal baths, other modality categories of medicinal plants include decoctions, teas, food and spices, tinctures, crushed or burnt, and externally applied. Decoctions are the second most common modality category (Table 4) with 106 species (34.6%). It is also one of the most common ways that traditional Chinese medicines are used. People usually use water to decoct the medicinal plant for a long time and finally take the decoction to treat certain illnesses. Making herbal tea and cooking is the third (79 spp., 25.8%) and fourth (44 spp., 14.4%) processing methods, respectively. It is noteworthy that the great majority of medicinal plants for food are used for infant malnutrition. More than 10% of the medicinal species are externally applied which is mostly for treating traumatic injuries. Local people usually crush these herbs and put on the wound to stop bleeding, diminish inflammation, relieve pain, and accelerate recovery.

**Table 4:** The modality of medicinal plants in the market in Jianghua

Modality	Records	Percentage	Modality	Records	Percentage
Medicinal bath	179	58.5	External use	36	11.8
Decoction	106	34.6	Tincture	11	3.6
Tea	79	25.8	Spice	2	–
Food	44	14.4	Burnt	1	–

Seeds of *Gleditsia sinensis* can be used incinerated to treat skin diseases like itching (it can also be used by decoction and medicinal bath). Several studies revealed that the chemical constituents extracted from *Gleditsia sinensis* showed good anti-bacterial, anti-allergy, anti-inflammatory, and anti-proliferative bioactivities [25–27]. The incineration process is unique: the local people typically use a flame to burn the *Gleditsia sinensis* seeds. Then, they hold a steel knife and make sure the blade is on the top of both the flame and the seeds to collect the soot, and it will be scraped off the blade and painted on the afflicted part of the patient. Besides the Yao people in Hunan Province, the Dong people in Guangxi Region also use this incineration method to treat illnesses. According to our previous study on the medicinal market in Guangxi Province (unpublished), the Dong people use the same method to incinerate certain poisonous plants like the root of *Alangium chinense*, the whole herb of *Macleaya cordata*, and the root of *Tripterygium wilfordii*. This method is thought to detoxify these poisonous plants, according to some Dong practitioners.

#### Frequency of occurrence and RI index of some medicinal plants

**Fig. 2:**
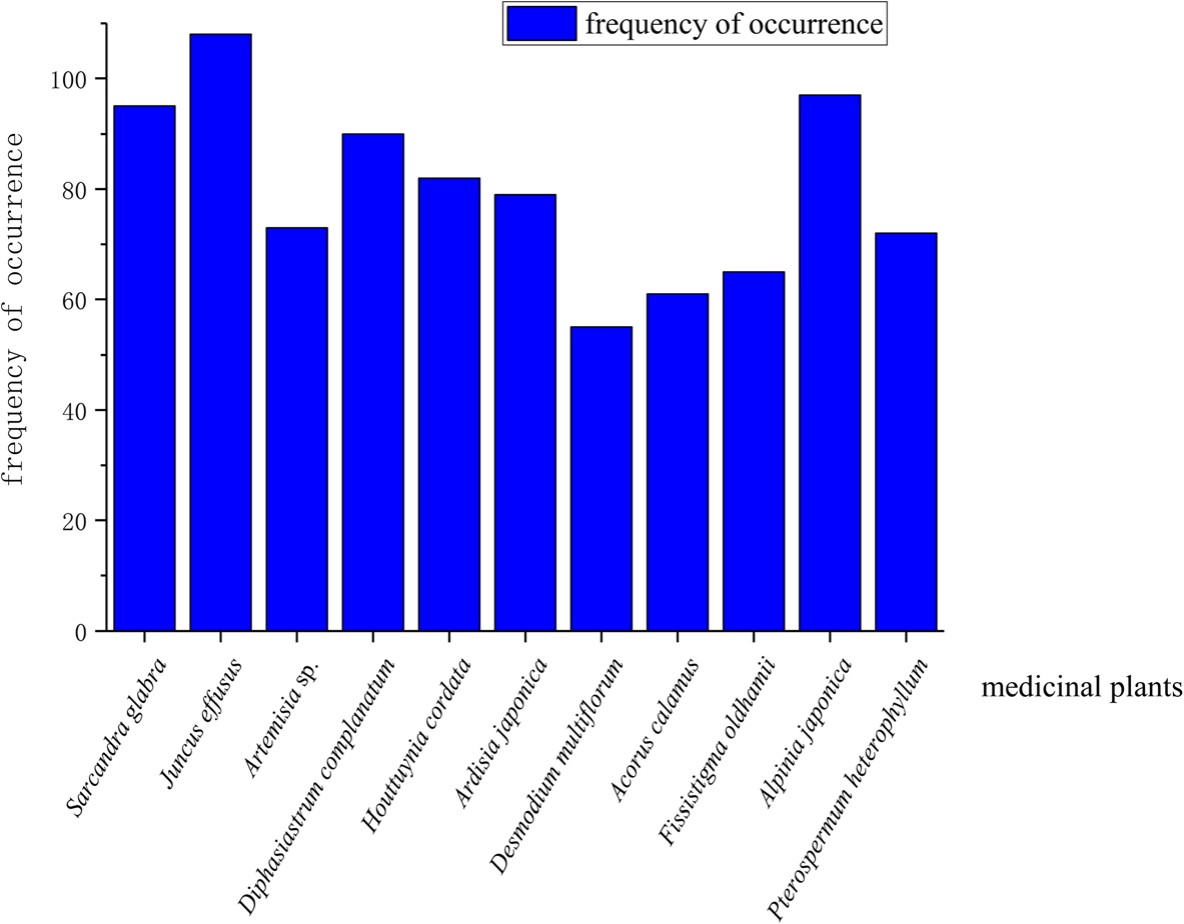
The frequency of occurrence of some medicinal plants in Jianghua County

The frequency of occurrence of each medicinal herb was recorded. This frequency signifies how many stalls sold a particular medicinal plant species. Some plant species were more frequently found than others (Fig. 2). Most species are used for clearing the inner heat and treating rheumatism. These results (Fig. 2) suggest that (1) the plants are widely distributed in local habitats and may be relatively easier to access; (2) the plants may have comparatively better effectiveness than the others so that they are more popular among local communities; and (3) inner heat and rheumatism are common problems for local people confirming the result from Table 3. High demands for those herbs and their effectiveness might be the major reasons leading to the high frequency of occurrence about the medicinal market.

The relative importance index is used to reflect the comprehensive utilization value [19]. The species with RI index greater than 0.4 are listed in Table 5. The modality types of these species are more various than other species. Most of them are edible and may be cooked as food and made into herbal tea or medicinal tincture. This character of being both edible and therapeutic indicated that these plants (Table 5) might be safer to humans with fewer side effects. Another reason for the relatively high RI index is that those species are easily acquired in local habitats and thereby make them more. The species themselves are locally widespread. For example, *Hedyotis auricularia*, *Cirsium japonicum*, and *Verbena officinalis* can be easily found on the roadsides and in the fields. *Gleditsia sinensis*, *Damnacanthus indicus*, and *Ardisia japonica* often appear in both wild and home gardens according to our observations. According to our interviews, almost everyone, including both vendors and local residents, can distinguish these species (Table 5). These species listed in Table 5 have high value in use with good potential for future development.

**Table 5:** The medicinal plants with higher RI

Name	Medicinal effectiveness type	Modalities	RI
*Cirsium japonicum* (Thunb.) Fisch. ex DC.	Nourishing, treating gynopathy, promoting blood circulation, stopping bleeding, eliminating inflammation	Decoction, external use, medicinal bath	0.49
*Verbena officinalis* L.	Treating rheumatism, treating venomous snake bite, heat clearing, promoting blood circulation, eliminating inflammation	Decoction, external use, medicinal bath	0.49
*Achillea millefolium* L.	Treating rheumatism, traumatic injury, treating gynopathy, snake bite	External use; decoction water; medicinal bath	0.47
*Rotala rotundifolia* (Buch.-Ham. ex Roxb.) Koehne	Heat clearing, traumatic injury, treating snake bite, skin disease	Decoction, external use, medicinal bath	0.47
*Pterospermum heterophyllum* Hance	Treating rheumatism, relaxing tendons and activating collaterals, relieving pain, treating arthritis,	Herbal tea, medicinal bath, food (stew with chicken)	0.47
*Agrimonia pilosa* Ledeb	Treating gastrointestinal disease, diminishing inflammation, stopping bleeding, treating heatstroke	Medicinal bath, medicine, herbal tea	0.47
*Artemisia dubia* Wall. ex Bess.	Treating rheumatism, heat clearing and detoxifying, diminishing inflammation, expelling parasite	Decoction, external use, medicinal bath	0.47
*Hedyotis auricularia* L.	Heat clearing and detoxifying, treating gastrointestinal disease, relieving cough, treating cold, promoting blood circulation, skin disease, snake bite	Herbal tea, medicinal bath	0.40
*Achillea millefolium* L.	Treating rheumatism, traumatic injury, treating gynopathy, snake bite	External use, decoction water, medicinal bath	0.47

### Demographics of vendors

Most vendors are Yao mountain people, and they can access many wild medicinal plants easily. However, in most cases, only elder vendors can speak the Yao language while the younger generation only speaks Mandarin Chinese or other local dialects because of the education and cultural fusion brought by the rapidly changing society and vigorous construction in the rural area. The age and gender of vendors have been recorded and analyzed (Table 6). The age range for vendors was 22–83 years old. The number of vendors older than 50 years old accounts for about 70% among all vendors. Those between 50 and 59 are the most with 90 (32.6%) people. The age composition for all vendors is slightly aging, but there are still many younger vendors, especially in 30–49 years old. Vendors younger than 30 years old are only 12 people (4.4%). This age composition reflects the succession problem of local traditional knowledge of Yao medicinal plants.

**Table 6:** The demographics of vendors

	20–29		30–39		40–49		50–59		60–69		70–79		> 80		Total
A	M	W	M	W	M	W	M	W	M	W	M	W	M	W	
B	8	4	20	8	24	14	44	46	16	28	32	32	0	2	276
C	12		28		36		90		44		64		2		276
D	4.35		10.14		13.04		32.61		15.94		23.19		0.73		100%

*A* ganders, *B* number of people, *C* number of people in different age groups, *D* percentages

As for the gender structure of the vendors, the number of men and women older than 50 years old is about equal. But under 50 years old, the number of men is twice the number of women. It is probably because that women dedicate themselves to housework, childcare, keeping livestock, and farmyard management while the men more commonly collect wild medicinal herbs in the high mountains. The interviews with the young vendors also showed that collecting the wild medicinal plants and selling them were considered only a temporary job. Much of the work collecting plants is done by the older generation and sold by the youngers who have other steady jobs. It was also found that the medicinal plants sold by elder vendors generally showed more botanical diversity but were gathered in relatively smaller amounts, while the plants sold by younger vendors were less diverse botanically but in larger amounts. These differences indicated that elder vendors master more traditional medicinal knowledge than younger vendors while younger vendors have more energy to search larger areas to collect larger amounts of wild medicinal plants. All these research findings suggest that the local traditional Yao medicine-associated knowledge is gradually decreasing.

The medicinal market in the Dragon Boat Festival in Jianghua is in a relatively large-scale venue with 269 stalls or vendors according to our investigations. Such a big traditional medicinal market appears at present time with well-developed Western medicine indicating that local people have a rich traditional knowledge of herbal medicine and depend upon it. However, most of the medicinal plants are not expensive, and the profit margin is slim. The fact that the vendors are still willing to come even if it is hard to collect the plants and time consuming suggests that they believe this is not only just for obtaining income but also following their tradition and even a way to celebrate the birthday of the Yao Medicinal Lord. As for the buyers, almost everyone in each age group knows a lot about medicinal plants. It demonstrates that the traditional knowledge of medicinal plants is widespread in the Yao community. The speed of the disappearance of related traditional knowledge gets much slower which is closely due to the medicinal markets in festival days which have played a great role of knowledge sharing in local community.

### Conservation of Yao medicinal knowledge

The traditional knowledge of Yao medicine is apparently decreasing. For instance, local people only have medicinal baths on the important festivals including the Dragon Boat Festival, the Double Ninth Festival, and the Panwang Festival nowadays. But they used to take a medicinal bath once a day in the past, according to local people. Less frequent practices will partially make it harder to keep such knowledge. The demographics of vendors and the incomplete vernacular names of medicinal plants also reflected this truth on other aspects. Even though a modern writing system of Yao language has been created, most of the Yao people in Jianghua still prefer spoken tradition since they receive Mandarin education beginning in primary school. The lack of a widely adopted writing system of the Yao language is a vulnerability for knowledge transfer [28].

As for the conservation of Jianghua traditional medicinal knowledge, the biggest challenge is apparently the shortage of professional personnel. One problem is that the Yao youth do not know enough about traditional Yao medicine and they are not confident about it [16]. By the impact of modern Western medicine, some local people prefer to use faster and more precise methods instead of their own traditional practices [29]. In addition, although the old masters of Yao medicine are dying out, the young people are not willing to study it or make it as a livelihood because it is not enough for feeding the family [15]. Nowadays, the Chinese government has recognized ethnomedicine and published a series of policies to support their protection and development after the foundation of the whole country [30, 31]. However, it is still urgent to cultivate more professional talents in the field of ethnomedicine by issuing more preferential policies and funds. It is necessary and helpful to normalize Yao doctors, to systemize the Yao medicinal theory, and to publish accompanying textbooks as well as other academic books.

The conservation of local medicinal plant resources is also quite important especially the conservation of rare and endangered plant species. The maintaining of the biodiversity is the material insurance for the development of relative traditional knowledge. Some endangered plant species were observed being traded about the Jianghua medicinal market like *Cibotium barometz*, *Alsophila spinulosa*, *Dendrobium officinale*, and *Semiliquidambar cathayensis* [32]. The stem and bark area of *Semiliquidambar cathayensis* is a very popular and effective traditional medicine for rheumatism locally. According to our surveys, the trading volume of *Semiliquidambar cathayensis* stem is large, and this plant material was all collected from the wild. Large-scale collection of plant resources will damage the local biodiversity and finally affect the stability of the local ecosystem.

The medicinal market in the Dragon Boat Festival in Jianghua County is a significant cultural event. Using its fame to develop tourism and attract businesses and investment may bring considerable money, but extreme care must be taken not to do any harm to local biodiversity and cultural diversity [33, 34].

In such a beneficial environment with the support by the government, it is an opportunity for local government agencies to improve better development of the medicinal market. Based on this investigation and others, the local government should consider protecting and developing the medicinal market to provide a better environment for vendors and buyers. The training of young personnel will strongly support the sustainable development of Yao medicine. In the meanwhile, the local government can also support the practitioners to exploit related by-products and apply for patents, even combining with poverty alleviation. Additionally, the local biodiversity and biological resources especially some endangered species should be protected by issuing conservation regulations or laws and by popularizing the green and sustainable awareness among local people.

## Conclusion

The herbal medicinal market is an important traditional activity celebrating the Dragon Boat Festival in Jianghua County, China. The formation and development of this special market is not only closely involved with local social history, but also local natural environment. As an herb trading site, this market plays an important role in the local community for medicinal knowledge exchange and heritage.

The result of our study showed the rich taxonomic diversity of medicinal plants and the diversity of their medicinal parts, medicinal uses, and modality categories. Based on our investigations, 306 species (belonging to 113 families and 249 genera) were recorded. The taxonomic distribution of those medicinal herbs clearly demonstrates the taxonomic diversity of the marketplaces. The whole plants have been used most frequently. Treating rheumatism and clearing inner heat are the most frequent symptoms addressed by these local healers. Medicinal baths are a special tradition in Jianghua County and account for the most common modality of the medicinal herbs. It is important to use modern scientific methods to verify the safety and efficacy of these traditional practices.

Although our analysis of the vendors reflected the predicament of losing traditional knowledge, some results are still promising like the species richness at the medicinal market, the local popularity of medicinal plant use, and the positive attitude to the traditional Yao medicine by local youths. These positive phenomena are associated with the medicinal market in some level, and it might provide a valuable reference for other places to sustainably develop local traditional medicine. The cultivation of relevant talents and maintaining the local biodiversity may be viable solutions to further develop traditional Yao medicine in Jianghua. Based on this investigation, and taking modern Yao culture into consideration, some proposals for improved construction, the protection of the medicinal market on the Dragon Boat Festival, and the traditional medicinal knowledge have been made.
